# Layer-specific gene expression in epileptogenic type II focal cortical dysplasia: normal-looking neurons reveal the presence of a hidden laminar organization

**DOI:** 10.1186/2051-5960-2-45

**Published:** 2014-04-15

**Authors:** Laura Rossini, Valentina Medici, Laura Tassi, Francesco Cardinale, Giovanni Tringali, Manuela Bramerio, Flavio Villani, Roberto Spreafico, Rita Garbelli

**Affiliations:** 1 Clinical Epileptology and Experimental Neurophysiology Unit, Istituto Neurologico “C. Besta”, Via Amadeo 42, 20133 Milano, Italy; 2 C. Munari Epilepsy Surgery Centre, Niguarda Hospital, Milan, Italy; 3 Department of Neurosurgery, Fondazione IRCCS, Istituto Neurologico “C. Besta”, Milan, Italy; 4 Department of Pathology, Niguarda Hospital, Milan, Italy

**Keywords:** Layer-specific genes, Focal cortical dysplasia, Epilepsy, Neuronal migration, Dysmorphic neurons

## Abstract

**Background:**

Type II focal cortical dysplasias (FCDs) are malformations of cortical development characterised by the disorganisation of the normal neocortical structure and the presence of dysmorphic neurons (DNs) and balloon cells (BCs). The pathogenesis of FCDs has not yet been clearly established, although a number of histopathological patterns and molecular findings suggest that they may be due to abnormal neuronal and glial proliferation and migration processes.

In order to gain further insights into cortical layering disruption and investigate the origin of DNs and BCs, we used *in situ* RNA hybridisation of human surgical specimens with a neuropathologically definite diagnosis of Type IIa/b FCD and a panel of layer-specific genes (LSGs) whose expression covers all cortical layers. We also used anti-phospho-S6 ribosomal protein antibody to investigate mTOR pathway hyperactivation.

**Results:**

LSGs were expressed in both normal and abnormal cells (BCs and DNs) but their distribution was different. Normal-looking neurons, which were visibly reduced in the core of the lesion, were apparently located in the appropriate cortical laminae thus indicating a partial laminar organisation. On the contrary, DNs and BCs, labelled with anti-phospho-S6 ribosomal protein antibody, were spread throughout the cortex without any apparent rule and showed a highly variable LSG expression pattern. Moreover, LSGs did not reveal any differences between Type IIa and IIb FCD.

**Conclusion:**

These findings suggest the existence of hidden cortical lamination involving normal-looking neurons, which retain their ability to migrate correctly in the cortex, unlike DNs which, in addition to their morphological abnormalities and mTOR hyperactivation, show an altered migratory pattern.

Taken together these data suggest that an external or environmental hit affecting selected precursor cells during the very early stages of cortical development may disrupt normal cortical development.

## Introduction

Focal cortical dysplasias (FCDs) are malformations of cortical development that are frequently associated with intractable epilepsy and are characterised by cortical dyslamination and abnormal cell morphology [1]. The most recent classification [2] recognises three types of FCD, with Type II being divided into two subtypes on the basis of the presence of dysmorphic neurons (DNs) alone (type IIa) or DNs together with balloon cells (BCs; Type IIb).

In both cases, cortical architecture is profoundly altered particularly within the core of the lesion, where DNs and normal-sized pyramidal cells co-exist with an overall reduction in neuronal density [3,4]. In addition to morphological changes (cytomegaly, abnormal dendritic arbors, and the loss of radial orientation), DNs are characterised by the aberrant expression of neuronal and glial markers with a prevalently neuronal-mature identity. BCs are also characterised by morphological anomalies such as voluminous cytoplasms, displaced nuclei and the co-expression of different mature/immature neuronal/glial markers with a prevalently glial-immature phenotype [5-7]. The lack of a clear cell-type specification may reflect alterations in normal neuronal and glial proliferation [2,8] and subsequent migration.

The cytological similarities between Type II FCD and tuberous sclerosis complex (TSC), a genetic disorder due to mutations in the *TSC1* or *TSC2* gene, and evidence of abnormal activation of the mammalian target of rapamycin (mTOR) signalling cascade, have led to the hypothesis that this pathway is involved in the pathogenesis of DNs and BCs [7,9,10], the origin of which is still debated. It has been suggested that they may arise from radial glial progenitor cells in the Ventricular Zone (VZ) [11], but it is still unclear whether they derived from a single or multiple progenitor cell, or whether they arise at a particular moment or at different times during the proliferation phase [12].

One means of gaining further insights into the structural abnormalities of dysplastic cortex and clarifying the cellular origin of DNs and BCs is to evaluate the expression patterns of an appropriate panel of layer-specific genes (LSGs). Despite their specific functions such as transcription factors, calcium-binding proteins, receptors, etc.), these genes have the distinctive characteristic of being expressed in (mainly glutamatergic) cortical neurons with very high laminar specificity, and can thus be used as markers to identify different cortical layers. This not only allows specific neuronal subpopulations to be identified on the basis of their expression profiles, but also provides information concerning their birth dates and migration [13,14]. Over the last few years, a number of studies have used LSGs to investigate cortical development under normal and pathological conditions in animal models [15-18] and humans [19-25].

The aim of this study was to investigate the expression of a panel of LSGs covering all cortical layers (*Cux2*, *Rorβ*, *Er81*, *Nurr1* and *CTGF)* in surgical specimens from a cohort of 20 patients with a neuropathological diagnosis of Type IIa/b FCD in order to learn more about cortical layering disruption and the origin of DNs and BCs.

## Methods

### Patient and specimen selection

We collected specimens from 20 patients who underwent surgery to treat intractable epilepsy at the C. Munari Epilepsy Surgery Centre of Niguarda Hospital and the Department of Neurosurgery of Besta Neurological Institute (both in Milan, Italy). The protocols of the pre-surgical investigation procedures were the same at both centres, and included high-resolution magnetic resonance imaging (MRI) and video-electroencephalography (VEEG) monitoring in order to define the epileptogenic zone and surgical strategy. The following individual MRI aspects were considered: focal thickening of the cortex, blurring of the grey/white matter junction, abnormal signal intensity in the cortex and subcortical white matter, tapering of white matter signal changes towards the ventricle (trasmantle sign). The C. Munari Centre also used invasive pre-surgical procedures (i.e. stereo-EEG) after appropriate discussions when the imaging and non-invasive electroclinical data were discordant.

All of the resections were performed for strictly therapeutic reasons after informed consent had been obtained. The pre-surgical procedures for selecting candidates for epilepsy surgery have been described in detail elsewhere [26].

All of the patients had a neuropathological diagnosis of Type II FCD. The cortical specimens were divided into two groups: Type IIa (8 cases) and Type IIb (12 cases). Table 1 summarises the patients’ main clinical features.

**Table 1:** Clinical and neuropathological findings

**ID**	**Age at seizure onset (years)**	**Age at surgery (years)**	**Monthly seizure frequency**	**Side/site of surgery**	**Neuro-pathological diagnosis**	**Outcome (Engel class)**
1	0	1	300	L/TO	FCD IIa	Ia
2	10	29	30	L/T	FCD IIa	IV
3	0	2	100	L/O	FCD IIa	Ia
4	0	13	60	L/O	FCD IIa	Ia
5	0	1	60	R/F	FCD IIa	Ia
6	1	9	600	R/FCP	FCD IIa	Ia
7	4	29	2	L/T	FCD IIa	Ia
8	0	31	60	L/TO	FCD IIa	Ic
9	0	25	30	R/TPO	FCD IIb	Ia
10	3	39	30	R/T	FCD IIb	Ia
11	2	4	300	L/P	FCD IIb	Ia
12	2	45	20	R/F	FCD IIb	Id
13	2	3	10	L/F	FCD IIb	IIb
14	4	22	40	L/T	FCD IIb	Ia
15	8	47	45	R/F	FCD IIb	III
16	10	14	100	R/F	FCD IIb	II
17	8	11	20	L/T	FCD IIb	Ia
18	0	13	1	R/F	FCD IIb	Ia
19	1	14	30	R/T	FCD IIb	Ia
20	6	46	75	L/P	FCD IIb	Ib

Abbreviations: *FCD* focal cortical dysplasia, *F* frontal, *ID* patient identification number, *L* left, *O* occipital, *P* parietal, *R* right, *T* temporal.

### Immunohistochemical procedures

After surgical resection, the specimens were fixed in 4% paraformaldehyde for 24–36 hours, and alternate 5 mm thick slabs were embedded in paraffin for routine neuropathology or cut into 50 μm thick serial sections by means of a vibratome (VT1000S, Leica, Heidelberg, Germany) and addressed for the present study. For the routine neuropathological investigations, 7 μm thick sections were stained with hematoxylin and eosin, thionin, Kluver-Barrera and Bielschowsky stains, and additional series were processed for immunohistochemistry (IHC). The vibratome sections were also stained with thionin and processed for IHC. The following primary antibodies were used: monoclonal antibodies against neuronal nuclear protein (NeuN, 1:3000, Chemicon, Temecula, CA, USA), pan-neuronal specific for non-phosphorylated neurofilaments (SMI311R, 1:1000, Covance, San Diego, CA, USA) and microtubule associated protein-2a and b (MAP2, 1:200, Neomarkers, Freemont, CA, USA) as neuronal markers; monoclonal antibody against glial fibrillary acidic protein (GFAP, 1:15000, Chemicon) as a glial marker; monoclonal antibody against intermediate filament protein vimentin (VIM, 1:3000, Dako, Carpinteria, CA, USA) as a marker of BCs; and rabbit monoclonal antibody against phospho-S6 ribosomal protein (pS6 Ser235/236, 1:500, Cell Signaling Technology, Beverly, MA, USA) as a marker indicative of mTOR pathway hyperactivation. The IHC procedures have been described in detail elsewhere [27].

### *In situ* hybridisation

In all cases, vibratome sections adjacent to the IHC sections were processed for *in situ* hybridisation (ISH) experiments designed to detect *Cux2*, *Rorβ*, *Er81*, *Nurr1* and *CTGF* mRNA expression. Free-floating sections were incubated with digoxigenin(DIG)-labelled riboprobes that specifically hybridise with each of the five mRNA of interest, and then with an alkaline phosphatase-conjugated anti-DIG antibody (1:1000, Roche Diagnostics Mannheim, Germany). The procedure and riboprobe characteristics have been described in detail elsewhere [16,20,22,28]. There were no apparent signals in control sections with the sense probes.

### Quantitative analysis of ISH signals

To estimate the reduction of normal-looking neurons, we counted the number of positive cells for the different LSG in a single section for each sample using Image Pro Plus 7.0 software (Media Cybernetics, Bethesta, MD, USA). For each LSG, we outlined at 10× objective two equal regions of interest (lesional area and, when present, adjacent perilesional area) including the specific layer. Using a 20× objective, all the immunopositive normal-looking neurons within this area were manually counted. The considered areas were: 1.44 mm^2^ for *Cux2*, 1.35 mm^2^ for *Rorβ*, 1.5 mm^2^ for *Er81* and 1.1 mm^2^ for *Nurr1.* Differences between lesional and perilesional areas were assessed by means of a *t* test, with p-values of <0.05 being considered significant.

### Double fluorescence ISH/IHC

It was not always possible to make a clear morphological distinction between LSG-positive DNs and BCs when BCs and giant round-shaped neurons coexisted in the same areas. In these cases, we used double fluorescence ISH/ICC combining *Rorβ*, *Er81* and *Nurr1* riboprobes with anti-SMI, anti-MAP2 (to identify DNs) and anti-vimentin antibodies (to identify BCs); the *Cux2* riboprobe was not used because of its low fluorescent hybridisation signal. After the ISH procedures, the sections were incubated overnight with a mixture of rabbit anti-DIG antibody (1:100000, Sigma, St. Louis, MO, USA) and mouse anti-SMI (1:300) or anti-MAP2 (1:300) or anti-Vim antibodies (1:300), and then incubated with biotinylated goat anti-rabbit antibody (1:200, Vector, Burlingame, CA, USA) and indocarbocyanine (Cy) 3-conjugated goat anti-mouse antibody (1:500, Jackson Immunoresearch Laboratories, West Grove, PA, USA) at room temperature for 60 minutes. Finally, the sections were incubated with streptavidine-HRP (1:1000, PerkinElmer, Boston, MA*,* USA) at room temperature for 30 minutes, and then with biotinil tyramide fluorescinated in amplification reagent (1:50, PerkinElmer) at room temperature for three minutes. The fluorescent sections were examined using a Nikon D-Eclipse C1 confocal laser scanning microscope (Nikon, Tokyo, Japan) equipped with an argon-ion laser system and mounted on a light inverted microscope Eclipse TE2000 (Nikon).

### *Er81* and *Nurr1* cortical layer thickness in perilesional cortex

Sections of five samples of temporal cortex taken from different patients and processed using the *Er81* and *Nurr1* riboprobes were analysed in order to evaluate possible changes in cortical distribution in the perilesional zone. The thickness of the layers was calculated on the basis of the extent of the neurons labelled by each riboprobe, and was expressed as a percentage of the total depth of the cortical ribbon. Cortical thickness was measured only in the linear portion of the cortex, far from gyri and sulci. One section per case was considered, and three measures of each marker were made using scanned images at 20× magnification (Scanscope; Aperio Technologies, Vista, CA, USA). The data were compared with those obtained in a previous study in witch six normal temporal cortices were evaluated [22], and the differences were assessed by means of a *t* test, with p-values of <0.05 being considered significant.

## Results

### Clinical aspects

Table 1 shows the patients’ clinical data. Their mean age at epilepsy onset was three years (range 0–10; SD 3) and mean seizure frequency at the time surgery was 95 per month (range 1–600; SD 145). Mean age at the time of surgery was 20 years (range 1–47; SD 15), when the mean duration of epilepsy was 16 years (range 1–43, SD 14). On the basis of the MRI diagnostic criteria recently described by Colombo *et al*. [29], the imaging investigation allowed a diagnosis of Type II FCD in 19 patients (95%), whereas only temporo-polar blurring was observed in one patient. Trasmantle sign was present in 12 patients. The mean follow-up was five years (range 1–13; SD 3). The surgical outcome was fairly good, with 16 patients in Engel class I (80%; seven with Type IIa FCD and nine with Type IIb); in particular, 13 patients are in Engel class Ia. The remaining four patients are in class II, III, and IV because of the incomplete resection of the lesion. Although the study population was small, the data concerning seizure outcome are in line with previous reports [29,30], thus suggesting that the good results were independent of the subtype and the site and side of the excised lesion.

### Neuropathological characteristics of FCD

**Figure 1:**
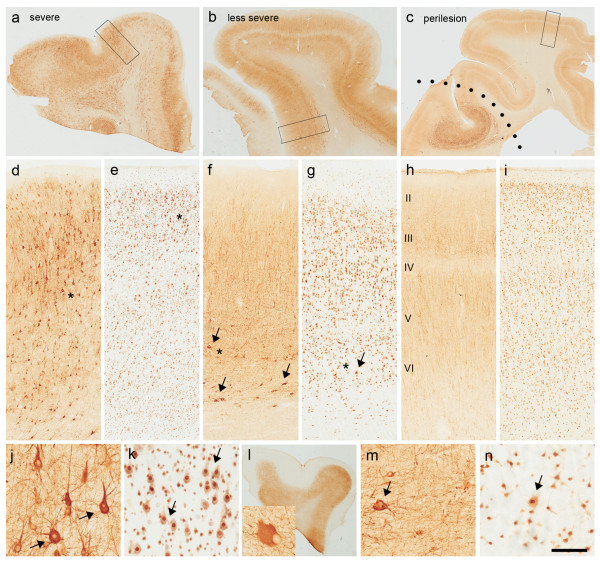
**Histopathological features of surgical samples taken from patients with Type IIb FCD. a**, **d**, **e**: Examples of a severe lesion (parietal lobe): **a**, **d** (SMI immunoreactivity, ir) and **e** (NeuN ir) highlight typical features of Type II FCD, which is characterised by the total disruption of cortical lamination and the presence of numerous DNs throughout the cortex intermingled with fewer normal-looking neurons. **j**, **k**: Details of the regions marked with asterisks in d and e, respectively, show DNs that are clearly distinguishable by their very large size and abnormal shape (arrows). In the same case, vimentin-positive BCs **(l)** are diffusely distributed in the subcortical white matter. **b**, **f**, **g**: Examples of a less severe lesion (frontal lobe). **b**, **f** (SMI ir) and **g** (NeuN ir) showing cortical areas in which lamination is relatively preserved and DNs (arrows) are scattered in restricted cortical layers (deep layers) intermingled with numerous normal-looking neurons. **m**, **n**: Details of the regions marked with asterisks in f and g, respectively, show DNs (arrow) and normal-looking neurons. **c**, **h**,** i**: Examples of perilesional cortex (temporal lobe). **c**, **h** (SMI ir) and **i** (NeuN ir) showing normal cortex adjacent to the principal lesion. Scale bars: 3.8 mm **(a)**; 2.5 mm** (b)**; 3.6 mm **(c)**; 540 μm **(d, e)**; 450 μm **(f, g)**; 414 μm **(h, i)**; 120 μm **(j, k, m, n)**; 4.68 mm **(l)** and 50 μm for inset in **l**.

The resections of the 20 patients (eight with Type IIa FCD and 12 with Type IIb) involved different cortical areas (see Table 1). The analysed specimen from 16 patients (seven with Type IIa and nine with Type IIb) was characterised by severe disruption of the laminar cortical structure. DNs were distributed throughout the cortical laminae except for layer I, sometime aggregated in neuronal clusters and, in six cases, were also present in the underlying white matter (Figure 1a,d,e). In four patients (one with Type IIa and three with Type IIb), the laminar structure was less severely affected, with fewer DNs mainly clustered in particular cortical layers (Figure 1b,f,g). However, it should be noted that other slabs from these cases used for histopathology rather than LSG detection showed profound disruption of the cortical architecture, suggesting the periphery of the lesion. Both the severely and less severely affected contained normal-looking neurons intermingled with DNs (Figure 1j,k,m,n). BCs (the hallmark of Type IIb FCD identified by means of vimentin immunopositivity) were always present in the white matter (Figure 1l), and dispersed throughout the cortex (in five cases) or clustered in layer I (three cases). Fifteen of the 20 specimens showed a normal perilesional cortex in which only rare and scattered DNs were detected (Figure 1c,h,i).

### Layer-specific gene expression in FCD

The lamina-specific expression patterns of the considered genes have been previously demonstrated in normal temporal and frontal cortex tissue: briefly, *Cux2* mRNA expression is observed in many cells in layers II and III; *Rorβ* mRNA is intensely expressed in layer IV and a few scattered cells in neighbouring layers; *Er81* and *Nurr1* mRNAs are respectively detected in neuronal subpopulations in layers V and VI; and *CTGF* mRNA is observed in a few cells in layer VI and the white matter [20,22]. An additional figure shows these features in detail (see Additional file 1).

**Figure 2:**
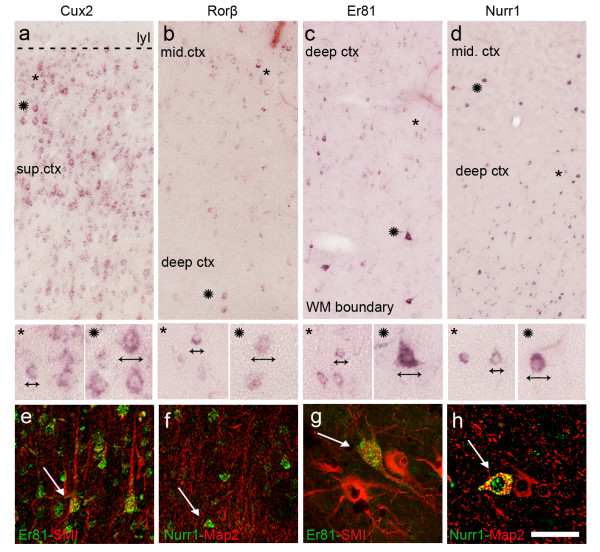
**Layer-specific gene (LSG) expression in normal-looking neurons and DNs. a**-**d**: Adjacent sections, obtained from a parietal lobe, showing regions expressing *Cux2***(a)**, *Rorβ***(b)**, *Er81***(c)** and *Nurr1***(d)**. In particular, areas from superficial cortex (sup.ctx), middle cortex (mid.ctx) and the boundary between the deep cortex and the white matter (wm) are shown. For each LSG, normal-looking neurons (asterisks) and DNs (stars) are easily distinguishable by their different size and shape (see high magnification images). Confocal double immunofluorescence images combining immunoreactivity for neuronal markers (SMI311 and MAP2) with ISH for *Er81* and *Nurr1* showed the morphology of the normal-looking neurons **(e, f)** and DNs **(g, h,)**. Scale bars: 200 μm **(a-d)** and 60 μm for high magnification images; 55 μm **(e-h)**.

In the pathological specimens used for this study, *Cux2*, *Rorβ*, *Er81* and *Nurr1* mRNAs were expressed in both abnormal and normal-looking neurons, which could be clearly distinguished on the basis of their differently sized bodies and nuclei (Figure 2a-d). *CTGF* mRNA expression was scanty and observed only in a few normal cells in the deep layers and in the white matter (data not shown).

**Figure 3:**
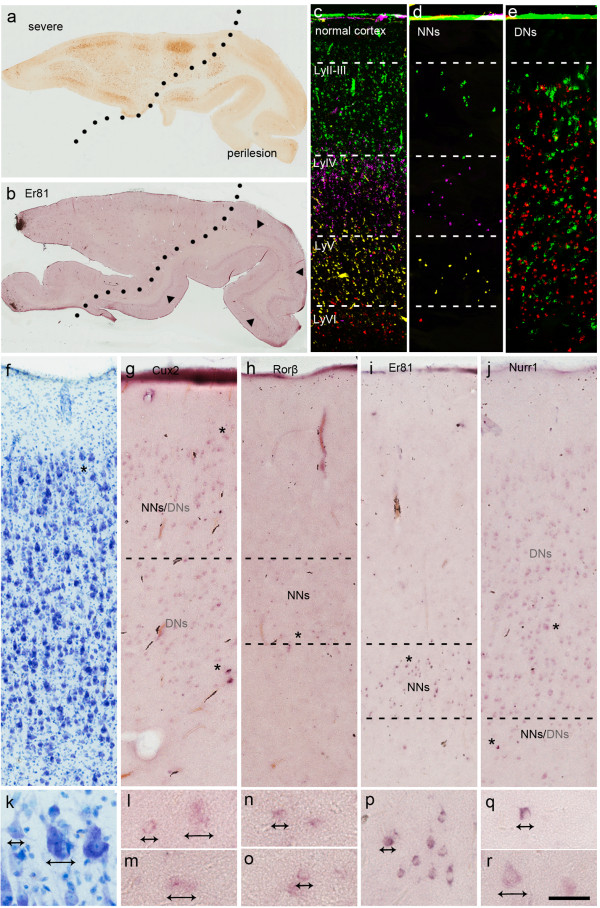
**LSG expression in Type II FCD (severe area).** Typical LSG expression in a case of Type IIa FCD (occipital lobe) histopathologically characterised by a severe lesion with an adjacent perilesional area (**a**, SMI ir). **b**: Example of *Er81* pattern showing intense expression in layer V of the perilesional cortex (arrowheads), and less intense and dispersed expression in the core of the lesion. **c**-**e**: Single ISH images of each gene in the severe lesion converted to pseudo-colour images (green for *Cux2*, fuchsia for *Rorβ*, yellow for *Er81*, and red for *Nurr1*) for overlay representation. The cortical distributions of normal-looking neurons (NNs, **d**) and DNs **(e)** are shown separately, and juxtaposed with normal cortex for purposes of comparison **(c)**. Note the normal laminar distribution and reduced number of NNs in comparison with the dispersion of DNs. **f**-**j**: Adjacent sections from the same case as that shown in **a** (SMI ir) and **f** (thionin staining), showing the expression of *Cux2***(g)**, *Rorβ***(h)**, *Er81***(i)** and *Nurr1***(j)** mRNAs in normal-looking neurons and DNs. **k**-**r**: Details of the regions marked with asterisks in **f**-**j** showing the morphological features of the NNs and DNs. Scale bars: 5.2 mm **(a, b)**; 240 μm **(c-e)**; 195 μm **(f-j)**; 50 μm **(k-r)***.*

**Figure 4:**
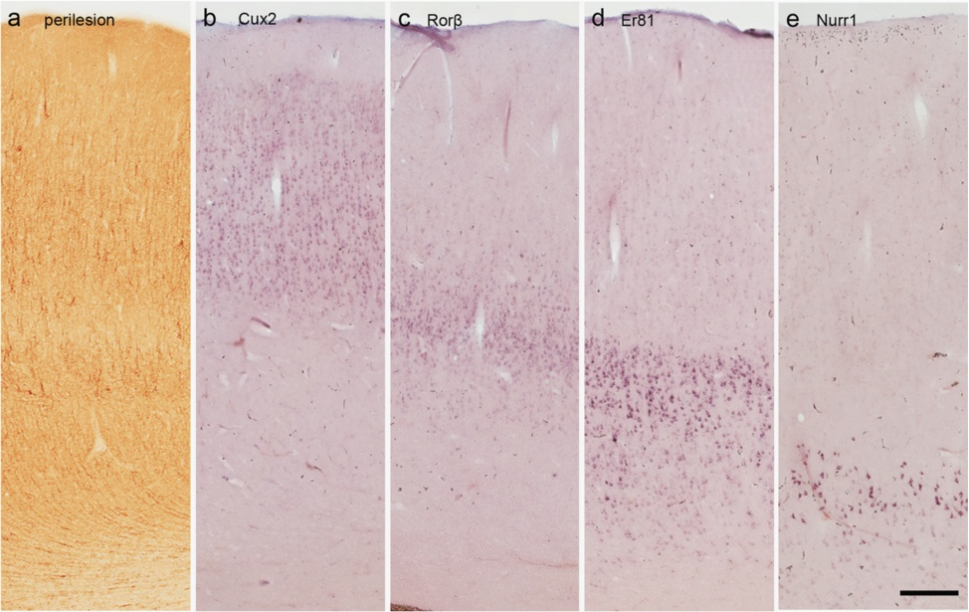
**LSG expression in perilesional cortex.** Typical LSG expression in perilesional cortex (frontal lobe) histopathologically characterised by a normal laminar organization and the absence of DNs (**a**, SMI ir). **b**-**e**: Adjacent sections from the same case as that shown in **a** showing the normal expression of *Cux2***(b)**, *Rorβ***(c)**, *Er81***(d)** and *Nurr1***(e)**. Scale bars: 295 μm **(a-e)**.

**Figure 5:**
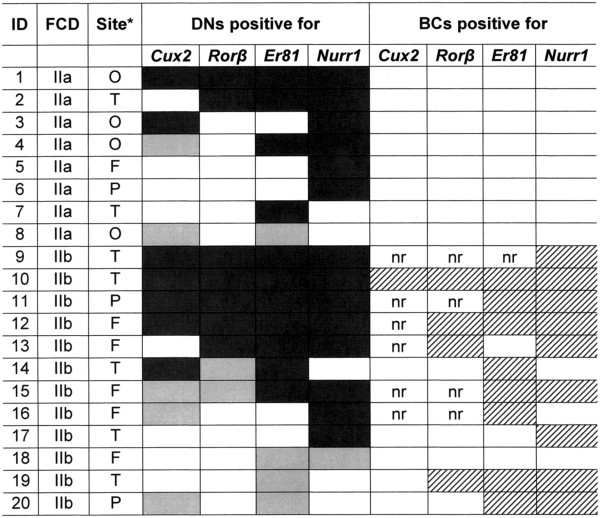
**Details of layer-specific gene expression in DNs and BCs.** For each case, the presence of DNs positive for the different LSG is indicated by a black or grey cell (the white cells indicate no labelling): the black cells indicate that DN distribution is severely affected, and the grey cells indicate DNs distributed in their corresponding layers. In the case of BCs, the hatched cells indicate the presence of the different LSGs, and the white cells indicate no labelling (nr = not clearly recognisable). Abbreviations: BCs: balloon cells; DNs: dysmorphic neurons; FCD: focal cortical dysplasia; ID: patient identification number. *The site is the cortical area that underwent ISH analysis (O: occipital; T: temporal; F: frontal; P: parietal).

In the 16 specimens showing severe laminar disorganisation and containing an adjacent perilesional area, there was a clear-cut difference in the distribution and signal intensity of gene expression moving from the core of the lesion toward the perilesional area (Figure 3a,b). Within the core of the lesion, normal-looking neurons (identified on the basis of their normal size and shape) were observed within the appropriate cortical laminae: *Cux2*-positive cells in the upper cortex, *Rorβ*-positive cells in the middle of the cortex, and *Er81*- and *Nurr1*-positive cells in the deeper cortex (Figure 3d,g-j,l,n-q), thus suggesting normal laminar organisation even in the core of the lesion (compare Figure 3d with 3c). Moreover, these neurons were visibly reduced in number in comparison with the adjacent perilesional area (particularly those that were positive for *Rorβ*), thus reflecting the reduced neuronal density inside the lesion (compare Figure 3g-j with Figure 4b-e). This data were further supported by quantitative analysis of normal-looking neurons cell density (see Additional file 2). DNs (identified on the basis of their very large size and altered morphology) showed the variable expression of all of the LSGs except for CTGF, without any laminar specificity (Figure 3g,j,l,m,r). The distribution of the DNs labelled by different LSGs was uneven regardless of the subtype of FCD (Type IIa *vs* IIb) and the lobar involvement of the dysplastic lesion. The details of LSG expression in each case are shown in Figure 5.

**Figure 6:**
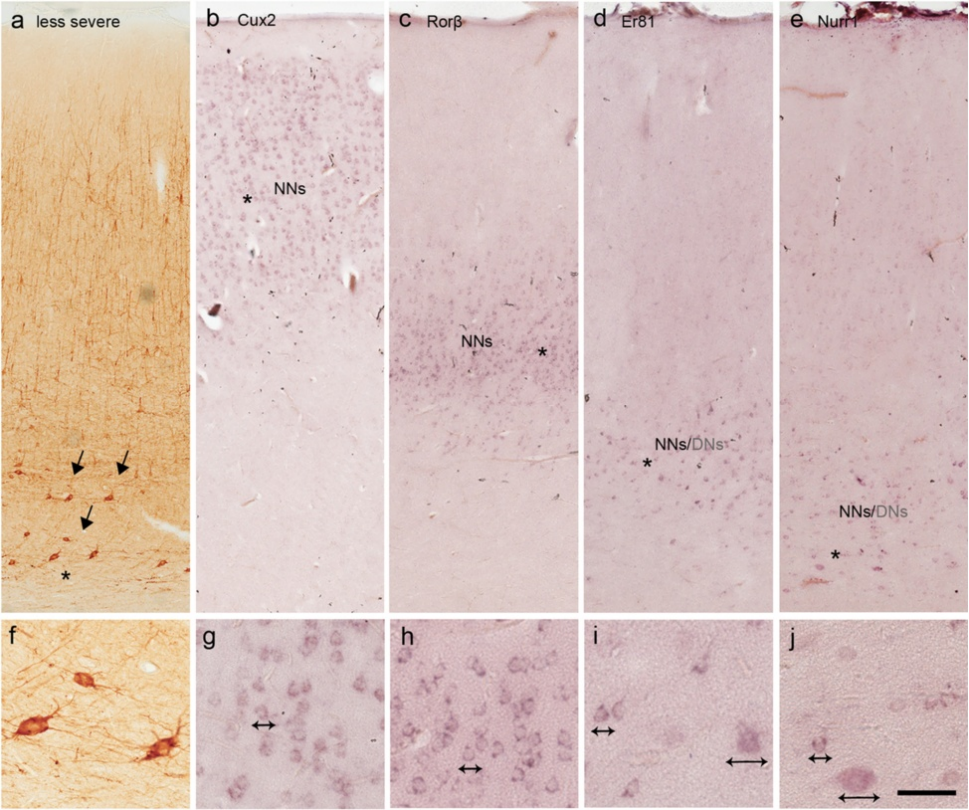
**LSG expression in Type II FCD (less severe area).** Typical LSG expression in a case of Type IIb FCD (frontal lobe) histopathologically characterized by a less severe lesion with DNs localised only in the deep portion of the cortex (**a**, SMI ir). **b**-**e**: Adjacent sections from the same case as that shown in **a** showing the expression of *Cux2***(b)**, *Rorβ***(c)**, *Er81***(d)** and *Nurr1***(e)**. Note that *Cux2* and *Rorβ* are expressed only in normal-looking neurons (NNs) in the appropriate cortical laminae **(b, c)**, whereas *Er81* and *Nurr1***(d, e)** are expressed in both NNs and DNs in the deep layers. **f**-**j**: Details of the regions marked with asterisks in **b**-**e** showing the morphological features of the NNs and DNs (arrows). Scale bars: 280 μm **(a-e)**; 90 μm **(f-j)***.*

In the four less severely affected specimens, the DNs were prevalently localised in layers III or V/VI (Figure 6a, f). The DNs labelled for *Cux2*, *Er81* and *Nurr1* (Figure 6d,e,i,j) retained their correct laminar position (unlike in the severely affected areas), and the normal-looking neurons intensely labelled with LSGs were properly distributed throughout the cortical thickness (Figure 6b-e, and g-j) and their density was similar to that observed in the perilesional cortex as confirmed by quantitative analysis (see Additional file 2).

In the 15 specimens that included perilesional cortex beside the core of the lesion, the laminar organisation was normal (Figure 4a) with the regular distribution of the LSGs in normal-looking neurons and no evidence of labelled DNs (Figure 4b-e). However, quantitative measurement of cortical thickness showed *Er81*-positive cells were more widespread throughout layer V than in the control cases (33,9 ± 8,1% for perilesional cortex versus 23,7 ± 3,8% for control cortex, p = 0,025).

### Layer-specific gene expression in BCs

**Figure 7:**
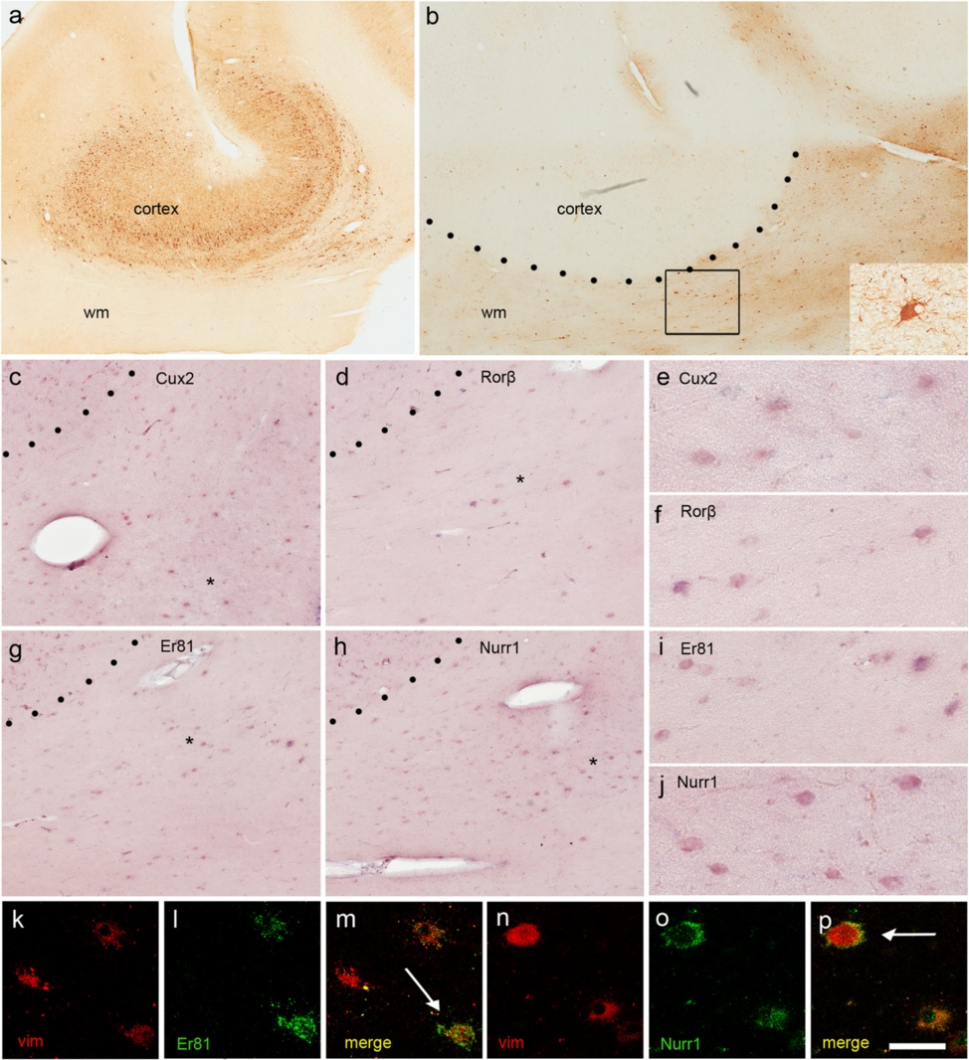
**Representative LSG expression in BCs. a**, **b**. Example of Type IIb FCD (temporal lobe) characterised by a severe lesion with numerous DNs dispersed throughout the cortex (**a**, SMI ir), and the presence of BCs in the white matter (**b**, vimentin ir). Adjacent sections from the same case as that shown in **a** and **b** showing the expression of *Cux2***(c)**, *Rorβ***(d)**, *Er81***(g)** and *Nurr1***(h)** in BCs in the white matter (boxed area in **b**). Note the heterogeneous expression of the LSGs in the BCs. **e**, **f**, **i**, **j**: Details of the regions marked with asterisks in **c**, **d**, **g** and **h** showing the morphological features of the BCs. Confocal double immunofluorescence images combining vimentin ir (red) with ISH for *Er81***(k-m)** and *Nurr1***(n-p)** confirmed the identification of the BCs. Scale bars:1, 41 mm **(a)**; 1,27 **(b)**; 357 μm **(c, d, g, h)**; 92 μm **(e, f, i, j)**; 70 μm **k**-**p**).

BCs were identified in 12 cases on the basis of their morphological features and vimentin immunolabelling, thus allowing a diagnosis of Type IIb FCD. They were mainly observed in the subcortical white matter (Figure 7a,b) but, in some cases, they were also distributed throughout the cortical thickness, and occasionally even invaded layer I. As in the case of the DNs, the *Cux2*, *Rorβ*, *Er81* and *Nurr1* riboprobes were variably expressed in BCs (Figure 7 c-j) with no specific labelling associated with their distribution in the different cortical layers or lobar involvement. These data were confirmed by the double fluorescent experiments using vimentin and the different LSGs (Figure 7k-p). Figure 5 shows the pattern of LSG expression in the BCs of each case.

### pS6 immunoreactivity in FCD

**Figure 8:**
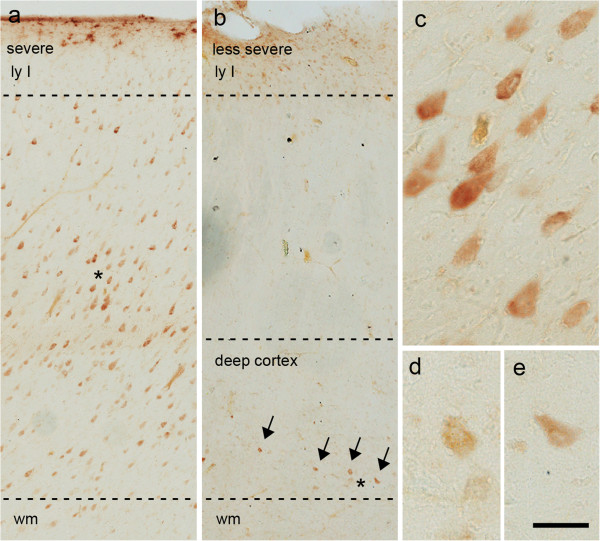
**Representative pS6 expression in Type II FCD.** Examples of pS6 expression in samples of severe **(a)** and less severe (**b**, arrows) Type II FCD; in both cases, the immunoreactivity suggests the presence of mTOR pathway hyperactivation in DNs. **c**-**e**. Details of the regions marked with asterisks in **a**, **b**. Scale bars: 268 μm **(a,b)**; 60 μm **(c,d,e)***.*

We observed immunoreactivity for pS6 in the DNs and BCs. Labelled DNs were detected in both the severely affected (Figure 8a,c) and less severely affected areas (Figure 8b,d,e), whereas no specific expression of pS6 was detected in the normal-looking neurons in either the core of lesion or the adjacent perilesional area.

## Discussion

Focal cortical dysplasias are among the most frequent brain malformations encountered in epileptic patients undergoing surgical treatment. In particular, Type II FCDs, which are often recognisable at the time of clinical screening, are characterised by an early onset of epilepsy, a high seizure frequency, and particular MRI and EEG (or SEEG) pictures [29-32], and surgical treatment often leads to seizure freedom even over long follow-up periods [30]. The main neuropathological features of Type II FCDs have been established since the original description by Taylor *et al*. [1], and so their classification has not varied in successive classification systems [2,33]. However, although many immunohistochemical studies have identified and characterised the various neuronal populations involved in Type II FCD, its aetiology and pathogenesis are unknown. The recent developmental and genetic classification of malformations of cortical development (MCD) published by Barkowich *et al*. [8] places Type II FCDs in the group of cortical malformations secondary to abnormal neuronal and glial proliferation, but the ontogenetic mechanisms and embryonic time window in which the derangement of cortical maturation and neuronal migration takes place remain unclear.

In order to gain more insights into the pathogenesis of cortical malformations, new LSG-based approaches have been proposed. Many different LSGs have been used in studies of animal and human specimens, and their expression profiles through the cortical layers have been defined; furthermore, it has been demonstrated that the expression and laminar location of most LSGs is preserved during phylogenesis and pre- and postnatal development [16-18,34]. On the basis of the assumption that, once specified during embryonic development, neuronal identity and laminar fate are apparently impervious to change even if cell migration is abnormal, it has been suggested that LSGs can be confidently used as markers to study cortical malformations [13,35]. However, although many studies have investigated different types of MCD by means of LSGs, only a few have specifically concentrated on Type II FCD [21,24,25,36].

The neuropathological hallmarks of Type II FCDs are disrupted cortical lamination, reduced cortical neuronal density, the presence of DNs and, in subtype IIb, the presence of BCs. In relation to these, the main findings of the present study are: i) although reduced in number, normal-looking neurons retain their correct laminar position even inside the core of the lesion, thus indicating a partial laminar organisation; ii) on the contrary, DNs and BCs are dispersed throughout the cortex and white matter without any laminar specificity, and show a highly variable LSG expression pattern; and iii) LSGs do not reveal any differences between Type IIa and IIb FCD.

The first and previously unknown finding is that the normal-looking neurons, albeit markedly fewer in comparison with the fairly normal adjacent cortex, can be found in the appropriate cortical laminae, which suggests that a certain proportion are properly generated and migrated. This implies that the genetic programme for neuronal migration is not disrupted *per se* and that the laminar disorganisation is mainly (if not exclusively) due to altered DN migration. Lower neuronal density in the region of the dysplasia than in the perilesional cortex is a consistent finding in Type II FCD, and may be due to different phenomena such as a local failure of cell proliferation or enhanced neuronal vulnerability in this area [3,4,37]; however, our data highlight for the first time that the number of normal-looking neurons is particularly reduced.

The second finding is that DNs are not only haphazardly displaced but (particularly within the core of the lesion) dispersed throughout the thickness of the cortex regardless of the LSG they express: for example, *Nurr1*-positive cells, which are normally found in the layer VI, were also present in the superficial part of the cortex, whereas *Cux2*-positive cells, which are normally found in layers II and III, were present in the depths of the cortical ribbon and white matter. Unfortunately, technical problems prevented us from using double-labelling in order to verify whether DNs express more than one LSG.

It is not possible to compare our findings with other published data concerning Type II FCD because the studies used different LSGs and different methodologies (IHC or ISH). Moreover, some published studies not specified the cell subtypes (i.e. normal-looking neurons *vs* DNs and BCs) expressing different LSGs [24,25]. Furthermore, as the LSGs we used are preserved during development, we could not verify the hypothesis that some DNs may retain the expression of genes that are exclusively present pre-natally as previously suggested [21,24,25].

Our finding that different LSGs are expressed in DNs improperly located in different layers of the cortex ribbon suggest that, whatever the defect, it occurred in the early period of development. Recent developmental data [38-41] indicate that radial glial cells (RGs) generate neurons by means of multiple rounds of self-renewal and asymmetric division, and that new-born neurons often use the parent cell’s radial fibre to migrate to the cortical plate [42]. Furthermore, it has been observed that primate corticogenesis is characterised by the appearance of a large sub-ventricular zone (SVZ) that seems to be important for the presence of resident progenitor cells that contribute to neuronal production and control neocortical size [43]. It has also been demonstrated that two sub-classes of human RGs co-exist in the ventricular zone (VZ) and the outer part of the SVZ (OSVZ), each of which act as neural stem cells in their respective locations and provide a mechanism favouring increased neuron production, which may be important for building a larger brain [44].

Although cortical neuron production begins in the VZ during gestational week 6 (GW6), the OSVZ does not arise until GW11, but then dramatically expands over the subsequent six GW to become the predominant germinal region in the neocortex. This suggests that OSVZ expansion and proliferation may increase the number of late-born neurons destined to became the future upper cortical layers, whereas the VZ generates the earliest-born neurons destined to form the deep cortical layers. On the basis of these developmental data, we can hypothesise that, during the early stages of cortical development (when the VZ is very actively proliferating and gives rise to the post-mitotic neurons that migrate to form the deep layers and the precursor cells for the SVZ), one hit is sufficient to modify the future lineage of some but not all progenitor cells in patients with Type II FCD.

Another finding of our study is that, like the DNs, the BCs expressed all of the LSGs regardless of their location and even they were found in all of the layers. These cells are immature, and their immunohistochemical profiles indicate the expression of vimentin, nestin and, in some cases, markers of precursor cells [6]. They seem to arise from radial cell precursors [11,21,45], but the fact that some of them co-express neuronal markers suggests aberrant cell-fate specification [5]. It is therefore conceivable that this abnormal differentiation may have been responsible for their labelling with our panel of LSGs, which are typically expressed in excitatory neurons. In any case, the fact that the distribution of LSGs in BCs was similar to that observed in DNs further supports the hypothesis that the primary hit inducing the malformation occurs during the early stages of development.

We did not identify any relationship between distinct patterns of LSG expression and FCD subtype or lobar involvement. The few published data in this regard are controversial insofar as some authors (using different LSGs) have found different distribution patterns and/or different numbers of immunopositive cells in Type IIa and IIb [24,25], whereas only Fauser *et al*. [36] have found differences in lobar LSG expression.

The processes involved in cortical development (including neurogenesis, cell migration and differentiation) are highly synchronised and coordinated by extra- and intracellular signals that converge on cytoskeletal remodelling [46,47], and it has been demonstrated that mutations in the genes encoding cytoskeletal proteins are involved in many malformations of cortical development [48]. Interestingly, one of the main histopathological features of Type II FCD is the presence of DNs characterised by the cytoplasmic engulfment of neurofilaments pushing the nucleus to the cell periphery and disrupting the regular distribution of chromatin. Similar DNs are found in patients with TSC in whom *TSC1* or *TSC2* gene mutations activate the mTOR cascade that affects many cell biological processes, including the regulation of actin dynamics [49].

In line with other recent findings [7,9,10], our data show that the DNs were labelled with pS6 antibody, which suggests that the mTOR pathway may also be involved in Type II FCD. As no genetic impairment has been found in this disease, it can be hypothesised that an “external” phenomenon could hit some of the precursor cells acting on the neuronal cytoskeletal framework very early during cortical development (when the VZ is proliferating), and that this leads to cytological alterations and the subsequently aberrant migratory stream. This would explain the presence of normal-looking neurons that show no changes in migration patterns.

In agreement with this hypothesis, the presence of a localized central nervous system infection during fetal brain development has been proposed as a new pathogenic mechanism for Type II FCD. In fact, recent studies have shown the presence of papillomavirus in BCs (but, surprisingly, not in DNs), and the presence of herpes and cytomegalovirus infections in both BC and DNs [50,51]. Moreover, human papillomavirus, the most common cause of cervical cancer in women, has been identified as an activator of the mTOR signalling cascade and the cytopathic effect of the infection on cervical epithelium results in balloon-like cells similar to those observed in Type II FCD. Although these data need further confirmation, they are in line with the hypothesis that an external or environmental hit affecting selected precursor cells during the very early stages of cortical development may interfere and disrupt normal cortical development as a result of mTOR activation.

One intriguing aspect of our findings is the presence of some DNs outside the core of the lesions (namely less severe lesion) that retained their correct laminar position in the cortical ribbon. Interestingly, like those associated with severe lesions, these DNs were also labelled with pS6 antibody. We do not have any definite explanation for this feature, but hypothesise that the high frequency of epileptic discharges characterising this form of dysplasia may induce a secondary pathological plasticity leading to morphological rearrangements, as suggested in various animal models [52].

## Conclusions

This study shows the existence of hidden cortical lamination involving normal-looking neurons, which retain their ability to migrate correctly in the cortex, unlike DNs which, in addition to their morphological abnormalities and mTOR hyperactivation, show an altered migratory pattern. BCs also expressed all of the LSGs regardless of their location. Taken together these data suggest that an external or environmental hit affecting selected precursor cells during the very early stages of cortical development may disrupt normal cortical development.

## Competing interest

The authors declares that they have no competing interests.

## Supplementary Material

Additional file 1 **LSG expression in normal human temporal cortex.** Description of data: Adjacent sections from the temporal cortex showing the typical expression of *Cux2* (b), *Rorβ* (c), *Er81* (d) and *Nurr1* (e) mRNAs. The laminar localization of each mRNA is determined by the adjacent NeuN-immunostained section and is indicated by Roman numerals (a). Scale bars: 385 μm (a-e). Click here for file

Additional file 2 **Normal-looking neurons cell density.** a: Histogram showing the cell density of normal-looking neurons (NNs) positive for LSG in samples characterised by severe disruption of the cortical structure; note the significant reduction of NNs in lesional versus perilesiona areas. Conversely, in less affected samples (b), no differenced in NNs cell density is reported in lesional versus perilesional areas. Statistical significance is indicated by asterisks. Click here for file

## References

[B1] Taylor DC Falconer MA Bruton CJ Corsellis JA Focal dysplasia of the cerebral cortex in epilepsy J Neurol Neurosurg Psychiatry 1971 2 369 387 10.1136/jnnp.34.4.369 5096551 PMC493805

[B2] Blumcke I Thom M Aronica E Armstrong DD Vinters HV Palmini A Jacques TS Avanzini G Barkovich AJ Battaglia G Becker A Cepeda C Cendes F Colombo N Crino P Cross JH Delalande O Dubeau F Duncan J Guerrini R Kahane P Mathern G Najm I Ozkara C Raybaud C Represa A Roper SN Salamon N Schulze-Bonhage A Tassi L The clinicopathologic spectrum of focal cortical dysplasias: a consensus classification proposed by an ad hoc Task Force of the ILAE Diagnostic Methods Commission Epilepsia 2011 2 158 174 10.1111/j.1528-1167.2010.02777.x 21219302 PMC3058866

[B3] Thom M Martinian L Sen A Cross JH Harding BN Sisodiya SM Cortical neuronal densities and lamination in focal cortical dysplasia Acta Neuropathol 2005 2 383 392 10.1007/s00401-005-1062-0 16151726

[B4] Alonso-Nanclares L Garbelli R Sola RG Pastor J Tassi L Spreafico R DeFelipe J Microanatomy of the dysplastic neocortex from epileptic patients Brain 2005 2 158 173 15548558 10.1093/brain/awh331

[B5] Englund C Folkerth RD Born D Lacy JM Hevner RF Aberrant neuronal-glial differentiation in Taylor-type focal cortical dysplasia (type IIA/B) Acta Neuropathol 2005 2 519 533 10.1007/s00401-005-1005-9 15877232

[B6] Sisodiya SM Fauser S Cross JH Thom M Focal cortical dysplasia type II: biological features and clinical perspectives Lancet Neurol 2009 2 830 843 10.1016/S1474-4422(09)70201-7 19679275

[B7] Yasin SA Latak K Becherini F Ganapathi A Miller K Campos O Picker SR Bier N Smith M Thom M Anderson G Helen Cross J Harkness W Harding B Jacques TS Balloon cells in human cortical dysplasia and tuberous sclerosis: isolation of a pathological progenitor-like cell Acta Neuropathol 2010 2 85 96 10.1007/s00401-010-0677-y 20352236

[B8] Barkovich AJ Guerrini R Kuzniecky RI Jackson GD Dobyns WB A developmental and genetic classification for malformations of cortical development: update 2012 Brain 2012 2 1348 1369 10.1093/brain/aws019 22427329 PMC3338922

[B9] Miyata H Chiang AC Vinters HV Insulin signaling pathways in cortical dysplasia and TSC-tubers: tissue microarray analysis Ann Neurol 2004 2 510 519 10.1002/ana.20234 15455398

[B10] Ljungberg MC Bhattacharjee MB Lu Y Armstrong DL Yoshor D Swann JW Sheldon M D’Arcangelo G Activation of mammalian target of rapamycin in cytomegalic neurons of human cortical dysplasia Ann Neurol 2006 2 420 429 10.1002/ana.20949 16912980

[B11] Lamparello P Baybis M Pollard J Hol EM Eisenstat DD Aronica E Crino PB Developmental lineage of cell types in cortical dysplasia with balloon cells Brain 2007 2 2267 2276 10.1093/brain/awm175 17711980

[B12] Hua Y Crino PB Single cell lineage analysis in human focal cortical dysplasia Cereb Cortex 2003 2 693 699 10.1093/cercor/13.6.693 12764046

[B13] Hevner RF Layer-specific markers as probes for neuron type identity in human neocortex and malformations of cortical development J Neuropathol Exp Neurol 2007 2 101 109 10.1097/nen.0b013e3180301c06 17278994

[B14] Molyneaux BJ Arlotta P Menezes JR Macklis JD Neuronal subtype specification in the cerebral cortex Nat Rev Neurosci 2007 2 427 437 10.1038/nrn2151 17514196

[B15] Ferrere A Vitalis T Gingras H Gaspar P Cases O Expression of Cux-1 and Cux-2 in the developing somatosensory cortex of normal and barrel-defective mice Anat Rec A: Discov Mol Cell Evol Biol 2006 2 158 165 16419078 10.1002/ar.a.20284

[B16] Watakabe A Ichinohe N Ohsawa S Hashikawa T Komatsu Y Rockland KS Yamamori T Comparative analysis of layer-specific genes in Mammalian neocortex Cereb Cortex 2007 2 1918 1933 10.1093/cercor/bhl102 17065549

[B17] Moroni RF Inverardi F Regondi MC Watakabe A Yamamori T Spreafico R Frassoni C Expression of layer-specific markers in the adult neocortex of BCNU-Treated rat, a model of cortical dysplasia Neuroscience 2009 2 682 691 10.1016/j.neuroscience.2008.12.064 19174181

[B18] Moroni RF Cipelletti B Inverardi F Regondi MC Spreafico R Frassoni C Development of cortical malformations in BCNU-treated rat, model of cortical dysplasia Neuroscience 2011 2 380 393 21130845 10.1016/j.neuroscience.2010.11.061

[B19] Ferland RJ Batiz LF Neal J Lian G Bundock E Lu J Hsiao YC Diamond R Mei D Banham AH Brown PJ Vanderburg CR Joseph J Hecht JL Folkerth R Guerrini R Walsh CA Rodriguez EM Sheen VL Disruption of neural progenitors along the ventricular and subventricular zones in periventricular heterotopia Hum Mol Genet 2009 2 497 516 18996916 10.1093/hmg/ddn377 PMC2722192

[B20] Garbelli R Rossini L Moroni RF Watakabe A Yamamori T Tassi L Bramerio M Russo GL Frassoni C Spreafico R Layer-specific genes reveal a rudimentary laminar pattern in human nodular heterotopia Neurology 2009 2 746 753 10.1212/WNL.0b013e3181af3397 19535771

[B21] Hadjivassiliou G Martinian L Squier W Blumcke I Aronica E Sisodiya SM Thom M The application of cortical layer markers in the evaluation of cortical dysplasias in epilepsy Acta Neuropathol 2010 2 517 528 10.1007/s00401-010-0686-x 20411268 PMC2923329

[B22] Rossini L Moroni RF Tassi L Watakabe A Yamamori T Spreafico R Garbelli R Altered layer-specific gene expression in cortical samples from patients with temporal lobe epilepsy Epilepsia 2011 2 1928 1937 10.1111/j.1528-1167.2011.03246.x 21883179

[B23] Saito T Hanai S Takashima S Nakagawa E Okazaki S Inoue T Miyata R Hoshino K Akashi T Sasaki M Goto Y Hayashi M Itoh M Neocortical layer formation of human developing brains and lissencephalies: consideration of layer-specific marker expression Cereb Cortex 2011 2 588 596 10.1093/cercor/bhq125 20624841

[B24] Arai A Saito T Hanai S Sukigara S Nabatame S Otsuki T Nakagawa E Takahashi A Kaneko Y Kaido T Saito Y Sugai K Sasaki M Goto Y Itoh M Abnormal maturation and differentiation of neocortical neurons in epileptogenic cortical malformation: unique distribution of layer-specific marker cells of focal cortical dysplasia and hemimegalencephaly Brain Res 2012 2 89 97 22759905 10.1016/j.brainres.2012.06.009

[B25] Sakakibara T Sukigara S Saito T Otsuki T Takahashi A Kaneko Y Kaido T Saito Y Sato N Kimura Y Nakagawa E Sugai K Sasaki M Goto Y Itoh M Delayed maturation and differentiation of neurons in focal cortical dysplasia with the transmantle sign: analysis of layer-specific marker expression J Neuropathol Exp Neurol 2012 2 741 749 10.1097/NEN.0b013e318262e41a 22805777

[B26] Tassi L Garbelli R Colombo N Bramerio M Lo Russo G Deleo F Milesi G Spreafico R Type I focal cortical dysplasia: surgical outcome is related to histopathology Epileptic Disord 2010 2 181 191 doi: 10.1684/epd.2010.0327 20659869 10.1684/epd.2010.0327

[B27] Garbelli R Munari C De Biasi S Vitellaro-Zuccarello L Galli C Bramerio M Mai R Battaglia G Spreafico R Taylor’s cortical dysplasia: a confocal and ultrastructural immunohistochemical study Brain Pathol 1999 2 445 461 10.1111/j.1750-3639.1999.tb00534.x 10416985 PMC8098203

[B28] Hirokawa J Watakabe A Ohsawa S Yamamori T Analysis of area-specific expression patterns of RORbeta, ER81 and Nurr1 mRNAs in rat neocortex by double in situ hybridization and cortical box method PLoS One 2008 2 e3266 10.1371/journal.pone.0003266 18815614 PMC2533703

[B29] Colombo N Tassi L Deleo F Citterio A Bramerio M Mai R Sartori I Cardinale F Lo Russo G Spreafico R Focal cortical dysplasia type IIa and IIb: MRI aspects in 118 cases proven by histopathology Neuroradiology 2012 2 1065 1077 10.1007/s00234-012-1049-1 22695739

[B30] Tassi L Garbelli R Colombo N Bramerio M Russo GL Mai R Deleo F Francione S Nobili L Spreafico R Electroclinical, MRI and surgical outcomes in 100 epileptic patients with type II FCD Epileptic Disord 2012 2 257 266 22963868 10.1684/epd.2012.0525

[B31] Tassi L Colombo N Garbelli R Francione S Lo Russo G Mai R Cardinale F Cossu M Ferrario A Galli C Bramerio M Citterio A Spreafico R Focal cortical dysplasia: neuropathological subtypes, EEG, neuroimaging and surgical outcome Brain 2002 2 1719 1732 10.1093/brain/awf175 12135964

[B32] Chassoux F Devaux B Landre E Turak B Nataf F Varlet P Chodkiewicz JP Daumas-Duport C Stereoelectroencephalography in focal cortical dysplasia: a 3D approach to delineating the dysplastic cortex Brain 2000 2 Pt 8 1733 1751 10908202 10.1093/brain/123.8.1733

[B33] Palmini A Najm I Avanzini G Babb T Guerrini R Foldvary-Schaefer N Jackson G Luders HO Prayson R Spreafico R Vinters HV Terminology and classification of the cortical dysplasias Neurology 2004 2 S2 S8 15037671 10.1212/01.wnl.0000114507.30388.7e

[B34] Wang WZ Hoerder-Suabedissen A Oeschger FM Bayatti N Ip BK Lindsay S Supramaniam V Srinivasan L Rutherford M Mollgard K Clowry GJ Molnar Z Subplate in the developing cortex of mouse and human J Anat 2010 2 368 380 10.1111/j.1469-7580.2010.01274.x 20727056 PMC2992414

[B35] Hevner RF Daza RA Rubenstein JL Stunnenberg H Olavarria JF Englund C Beyond laminar fate: toward a molecular classification of cortical projection/pyramidal neurons Dev Neurosci 2003 2 139 151 10.1159/000072263 12966212

[B36] Fauser S Haussler U Donkels C Huber S Nakagawa J Prinz M Schulze-Bonhage A Zentner J Haas CA Disorganization of neocortical lamination in focal cortical dysplasia is brain-region dependent: evidence from layer-specific marker expression Acta Neuropathol Commun 2013 2 47-5960-1-47 24252438 10.1186/2051-5960-1-47 PMC3893528

[B37] Muhlebner A Coras R Kobow K Feucht M Czech T Stefan H Weigel D Buchfelder M Holthausen H Pieper T Kudernatsch M Blumcke I Neuropathologic measurements in focal cortical dysplasias: validation of the ILAE 2011 classification system and diagnostic implications for MRI Acta Neuropathol 2012 2 259 272 10.1007/s00401-011-0920-1 22120580

[B38] Lui JH Hansen DV Kriegstein AR Development and evolution of the human neocortex Cell 2011 2 18 36 10.1016/j.cell.2011.06.030 21729779 PMC3610574

[B39] Bystron I Blakemore C Rakic P Development of the human cerebral cortex: Boulder Committee revisited Nat Rev Neurosci 2008 2 110 122 10.1038/nrn2252 18209730

[B40] Hansen DV Lui JH Parker PR Kriegstein AR Neurogenic radial glia in the outer subventricular zone of human neocortex Nature 2010 2 554 561 10.1038/nature08845 20154730

[B41] Fietz SA Kelava I Vogt J Wilsch-Brauninger M Stenzel D Fish JL Corbeil D Riehn A Distler W Nitsch R Huttner WB OSVZ progenitors of human and ferret neocortex are epithelial-like and expand by integrin signaling Nat Neurosci 2010 2 690 699 10.1038/nn.2553 20436478

[B42] Noctor SC Flint AC Weissman TA Dammerman RS Kriegstein AR Neurons derived from radial glial cells establish radial units in neocortex Nature 2001 2 714 720 10.1038/35055553 11217860

[B43] Haubensak W Attardo A Denk W Huttner WB Neurons arise in the basal neuroepithelium of the early mammalian telencephalon: a major site of neurogenesis Proc Natl Acad Sci U S A 2004 2 3196 3201 10.1073/pnas.0308600100 14963232 PMC365766

[B44] Lukaszewicz A Savatier P Cortay V Giroud P Huissoud C Berland M Kennedy H Dehay C G1 phase regulation, area-specific cell cycle control, and cytoarchitectonics in the primate cortex Neuron 2005 2 353 364 10.1016/j.neuron.2005.06.032 16055060 PMC1890568

[B45] Cepeda C Andre VM Levine MS Salamon N Miyata H Vinters HV Mathern GW Epileptogenesis in pediatric cortical dysplasia: the dysmature cerebral developmental hypothesis Epilepsy Behav 2006 2 219 235 10.1016/j.yebeh.2006.05.012 16875879

[B46] Heng JI Chariot A Nguyen L Molecular layers underlying cytoskeletal remodelling during cortical development Trends Neurosci 2010 2 38 47 10.1016/j.tins.2009.09.003 19837469

[B47] Reiner O Sapir T Polarity regulation in migrating neurons in the cortex Mol Neurobiol 2009 2 1 14 10.1007/s12035-009-8065-0 19330467

[B48] Guerrini R Dobyns WB Barkovich AJ Abnormal development of the human cerebral cortex: genetics, functional consequences and treatment options Trends Neurosci 2008 2 154 162 10.1016/j.tins.2007.12.004 18262290

[B49] Costa-Mattioli M Monteggia LM mTOR complexes in neurodevelopmental and neuropsychiatric disorders Nat Neurosci 2013 2 1537 1543 10.1038/nn.3546 24165680

[B50] Chen J Tsai V Parker WE Aronica E Baybis M Crino PB Detection of human papillomavirus in human focal cortical dysplasia type IIB Ann Neurol 2012 2 881 892 10.1002/ana.23795 23280839

[B51] Liu S Lu L Cheng X Xu G Yang H Viral infection and focal cortical dysplasia Ann Neurol 2013 doi: 10.1002/ana.24037 10.1002/ana.24037 24122971

[B52] Colciaghi F Finardi A Frasca A Balosso S Nobili P Carriero G Locatelli D Vezzani A Battaglia G Status epilepticus-induced pathologic plasticity in a rat model of focal cortical dysplasia Brain 2011 2 2828 2843 10.1093/brain/awr045 21482549

