# Supplementary figures and images for: Layer-specific gene expression in epileptogenic type II focal cortical dysplasia: normal-looking neurons reveal the presence of a hidden laminar organization
Source: Acta Neuropathol Commun. 2014 Apr 15;2:45. doi: 10.1186/2051-5960-2-45 (PMC4023625; doi:10.1186/2051-5960-2-45)

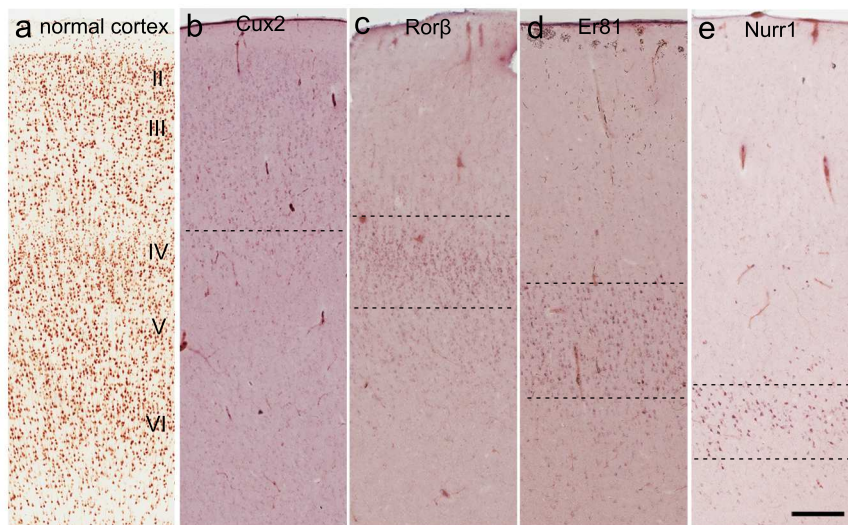

Additional file 1

Supplement: Additional file 1 — LSG expression in normal human temporal cortex. Description of data: Adjacent sections from the temporal cortex showing the typical expression of Cux2 (b), Rorβ (c), Er81 (d) and Nurr1 (e) mRNAs. The laminar localization of each mRNA is determined by the adjacent NeuN-immunostained section and is indicated by Roman numerals (a). Scale bars: 385 μm (a-e). [file 2051-5960-2-45-S1.pdf]

**A**

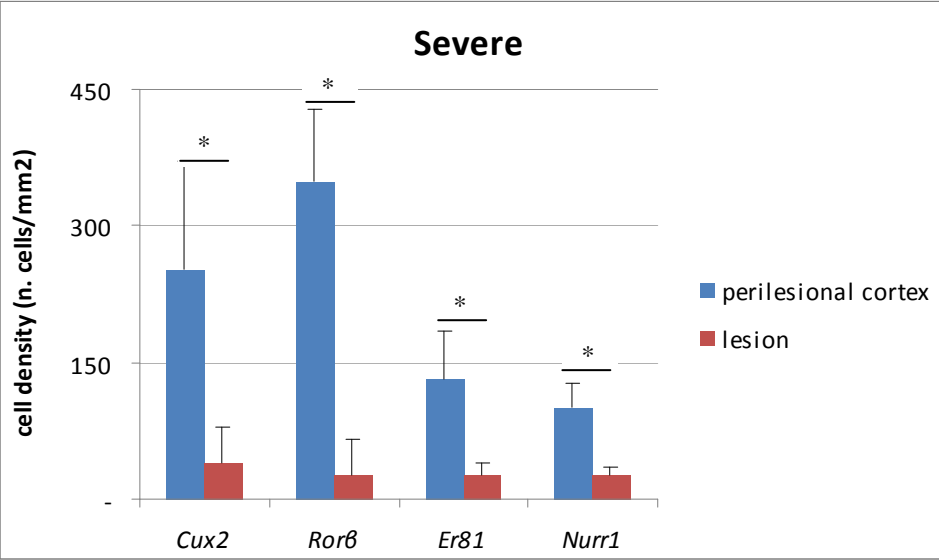

**B**

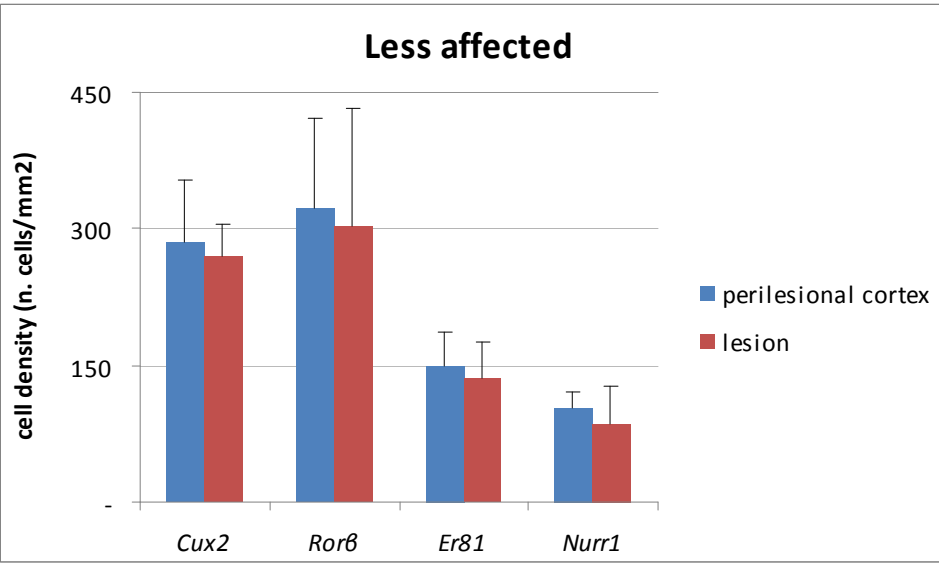

Supplement: Additional file 2 — Normal-looking neurons cell density. a: Histogram showing the cell density of normal-looking neurons (NNs) positive for LSG in samples characterised by severe disruption of the cortical structure; note the significant reduction of NNs in lesional versus perilesiona areas. Conversely, in less affected samples (b), no differenced in NNs cell density is reported in lesional versus perilesional areas. Statistical significance is indicated by asterisks. [file 2051-5960-2-45-S2.pdf]
